# Predicting the Risk of Alzheimer’s Disease and Related Dementia in Patients with Mild Cognitive Impairment Using a Semi-Competing Risk Approach

**DOI:** 10.3390/informatics10020046

**Published:** 2023-05-30

**Authors:** Zhaoyi Chen, Yuchen Yang, Dazheng Zhang, Jingchuan Guo, Yi Guo, Xia Hu, Yong Chen, Jiang Bian

**Affiliations:** 1 Department of Health Outcomes and Biomedical Informatics, University of Florida, Gainesville, FL 32611, USA; 2 Department of Biostatistics, Epidemiology and Informatics, University of Pennsylvania, Philadelphia, PA 19104, USA; 3 Department of Pharmaceutical Outcomes & Policy, University of Florida, Gainesville, FL 32611, USA; 4 Department of Computer Science, Rice University, Houston, TX 77005, USA

**Keywords:** Alzheimer’s disease, electronic health record, competing risks

## Abstract

Alzheimer’s disease (AD) and AD-related dementias (AD/ADRD) are a group of progressive neurodegenerative diseases. The progression of AD can be conceptualized as a continuum in which patients progress from normal cognition to preclinical AD (i.e., no symptoms but biological changes in the brain) to mild cognitive impairment (MCI) due to AD (i.e., mild symptoms but not interfere with daily activities), followed by increasing severity of dementia due to AD. Early detection and prediction models for the transition of MCI to AD/ADRD are needed, and efforts have been made to build predictions of MCI conversion to AD/ADRD. However, most existing studies developing such prediction models did not consider the competing risks of death, which may result in biased risk estimates. In this study, we aim to develop a prediction model for AD/ADRD among patients with MCI considering the competing risks of death using a semi-competing risk approach.

## 1. Introduction

Alzheimer’s disease (AD) and AD-related dementias (AD/ADRD) are a group of progressive neurological diseases. As the most common cause of dementia, AD accounts for 60% to 80% of dementia cases [1]. AD/ADRD poses significant public health burdens in the United States (US). It is estimated that there are 6.5 million adults over 65 years living with AD, with the number expected to reach 12.7 million by the year 2050 [1]. The estimated total healthcare cost for AD treatment in 2020 is estimated at USD 305 billion, with the cost expected to increase to more than USD 1 trillion as the population ages [2].

The progression of AD/ADRD can be conceptualized as a continuum in which patients progress from normal cognition to preclinical AD/ADRD (i.e., no symptoms but biological changes in the brain) to mild cognitive impairment (MCI) due to AD/ADRD (i.e., mild symptoms but not interfere with daily activities), followed by increasing severity of dementia due to AD/ADRD [1]. As an early stage of memory or other cognitive ability loss, MCI has usually been considered a pre-dementia phase of AD/ADRD. However, not all patients with MCI will transition to AD/ADRD dementia. Prior evidence suggests the existence of heterogeneity in AD progression pathways (e.g., faster progression or with different clinical syndromes) [3,4]. Characterizing and predicting different AD/ADRD progression pathways and the associated risk factors is a crucial step in understanding the mechanism of AD/ADRD.

It is estimated that about 10–15% of patients with MCI will transition to AD/ADRD each year, and after six years of follow-up, approximately 80% of MCI patients will be converted to AD/ADRD [5–10]. Therefore, early detection—thus, prediction models—of the transition of MCI to AD/ADRD are needed. There has been a considerable increase in efforts over the past few years to build machine-learning-based models for AD/ADRD prediction with clinical data such as neurobehavioral status exam scores, patient demographics, neuroimaging data, and laboratory test values [11–13]. Meanwhile, the proliferation of clinical research networks with large collections of real-world data (RWD), including electronic health records (EHRs), claims, and billing data among others, offers unique opportunities to generate real-world evidence (RWE) [14] that will have direct translational impacts on AD/ADRD research. Recent advancements in machine learning (ML) have led to success in various RWD analysis tasks, such as clinical risk prediction [15,16], disease subphenotyping [17,18], and personalized treatment [19]. Analyses of EHRs are complicated due to large sample sizes, high dimensionality, sparsity, and heterogeneity [20], but more importantly, an appropriate study design that accounts for the various potential biases inherently exists in observational EHR data.

A recent systematic review examined studies that used machine learning methods and clinical data to model risk for the progression of AD/ADRD [11]. Of the 64 papers included in the systematic review, about half of them modeled the development of AD/ADRD in individuals who were initially cognitively normal or had only MCI. However, most existing studies developing such prediction models do not consider the competing risks of other factors, such as death. Competing risks refer to the situation where the study population is at risk for more than one type of possibly correlated failure events [21], and it could lead to biased results and misleading interpretation of the hazard ratio if we simply treat death as random censoring and fit a standard Cox proportional regression model in the sense that it does not account for the scenario that a patient who experienced the AD/ADRD subsequently had death. In the case of AD/ADRD prediction, the study population is subject to both the risk of AD/ADRD and the risk of death. On one hand, individuals with MCI are at an increased risk of developing AD/ADRD; on the other hand, these individuals are also at an increased risk of death compared to those without MCI because of age and aging-related health conditions such as cardiovascular disease, diabetes, and cancer are more common in older adults. These conditions can cause long-term damage to the body’s systems, making individuals more vulnerable to complications and infections. Additionally, age-related changes in the immune system can weaken the body’s defenses against infections and increase the risk of mortality from infectious diseases. Thus, older adults are at an increased risk of death due to the accumulation of aging-related health conditions and their impact on overall health and immune function [22–25].

**Figure 1.**
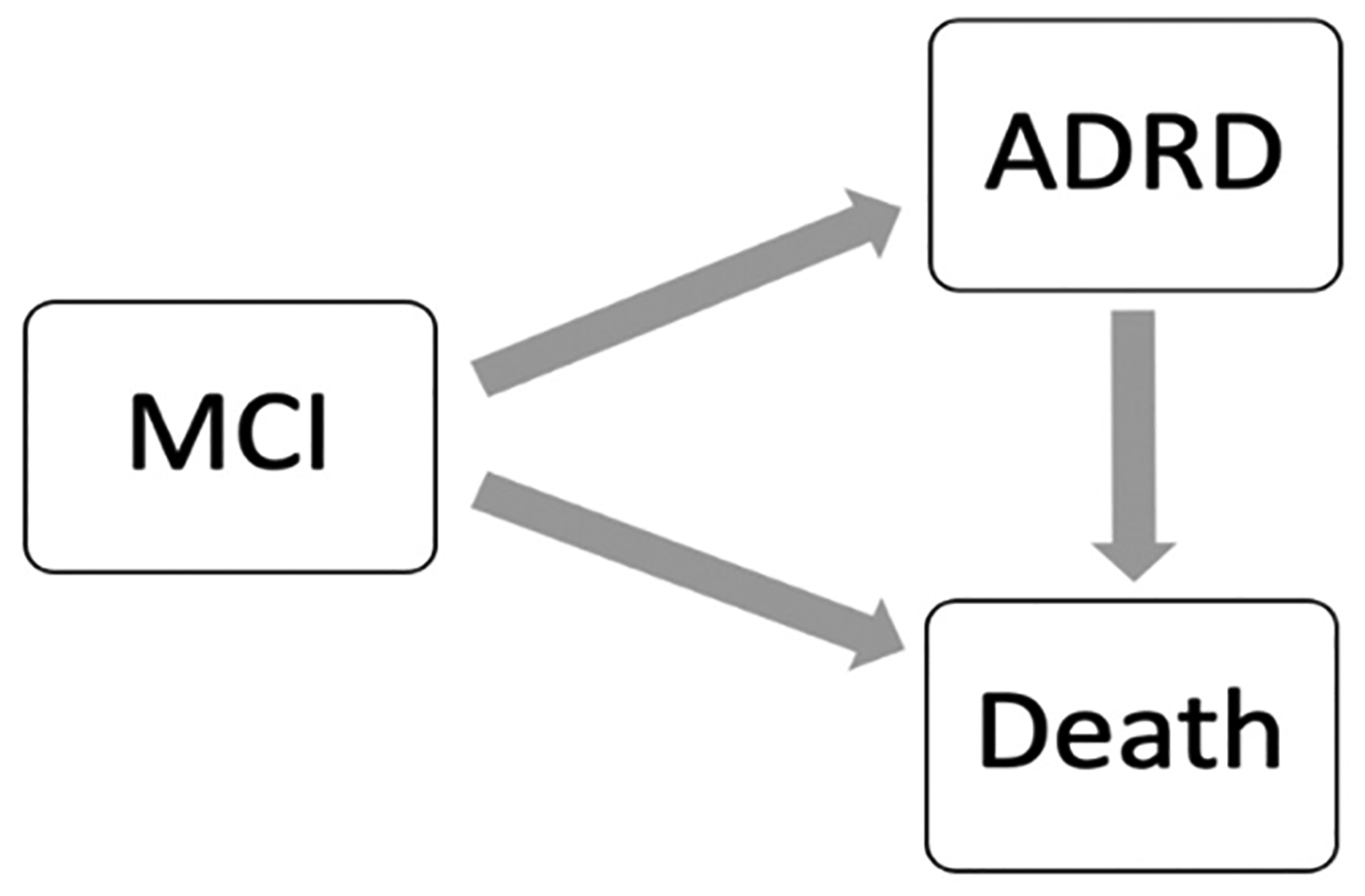
The illness–death process for semi-competing risks data.

The risk of death is a competing risk for the development of AD/ADRD, as individuals who die before developing AD/ADRD will not contribute to the incidence of AD/ADRD, meaning that the competing risk of death would censor the AD/ADRD outcomes; thus, the risk of death serves as an informative censoring for AD/ADRD failure events. Failure to account for death as a competing risk may lead to biased estimates of the incidence of AD/ADRD and inaccurate predictions of the risk of developing AD/ADRD. The data used in such prediction task can be viewed as semi-competing risks data with a non-terminal event (i.e., AD/ADRD) and a terminal event (i.e., death) [26], and the disease process can then be described as an illness–death process with three states: MCI, AD/ADRD, and death. The illness–death process is characterized by three hazard functions to quantify the transition rates between states, i.e., the hazard from MCI to AD/ADRD, the hazard from MCI to death, and the hazard from AD/ADRD to death, as shown in Figure 1. For simplicity, in this paper, we only consider the progression from MCI to AD/ADRD or death and aim to develop a prediction model for AD/ADRD among patients with MCI considering the competing risks of death using a semi-competing risk approach, while other semi-competing risks of AD/ADRD can be modeled similarly.

## 2. Materials and Methods

In this study, we used the structured electronic health records (EHR) data from the OneFlorida+ Clinical Research Network [27], one of the eight clinical data research networks contributing to the national Patient-Centered Clinical Research Network (PCORnet) funded by the Patient-Centered Outcomes Research Institute (PCORI). The OneFlorida+ network contains robust longitudinal and linked patient-level real-world clinical data of ~16.8 million Floridians, including data from Medicaid and Medicare claims, tumor registries, vital statistics, and EHRs from its clinical partners. The OneFlorida+ data is a Health Insurance Portability and Accountability Act of 1996 (HIPAA) limited data set (i.e., dates are not shifted) that contains detailed patient demographics and their clinical characteristics, including encounters, diagnoses, procedures, vitals, medications, and labs, following the PCORnet Common Data Model (CDM) [27,28]. The data contributed to the OneFlorida+ network undergoes rigorous quality checks and a privacy-preserving record linkage process is used to link and deduplicate patient records from multiple health systems and data sources (i.e., through Datavant required by PCORnet).

**Figure 2.**
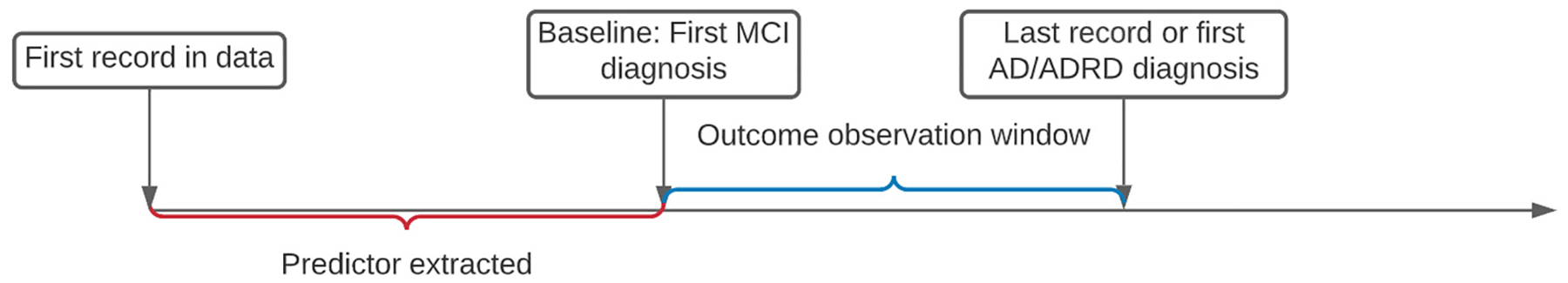
Patient timeline of the predictor extraction time window and the outcome observation window.

From the OneFlorida+ data, patients who had MCI diagnosis recorded in at least one inpatient or two outpatient encounters within a year were identified with ICD codes (ICD-9: 331.83, 294.9; ICD-10: G31.84, F09). For each MCI patient, their first MCI diagnosis was considered as the baseline, and the patients were followed-up until their first AD/ADRD diagnosis, death, or the last record available. Figure 2 displays the overall patient timeline of a typical patient.

For outcome identification (AD/ADRD diagnosis), we considered five conditions: Alzheimer’s disease (ICD-9: 331.0; ICD-10: G30, G30.0, G30.1, G30.8, G30.9), vascular dementia (ICD-9: 290.4, 290.40, 290.41, 290.42, 290.43; ICD-10: F01, F01.5, F01.50, F01.51), Lewy body dementia (ICD-9: 331.82; ICD-10: G31.83), frontotemporal dementia (ICD-9: 331.1, 331.11, 331.19; ICD-10: G31.0, G31.01, G31.09), and mixed dementias (i.e., multiple types of AD/ADRD dementias). A set of predictors were identified from the literature and extracted from each patient’s medical records prior to the baseline [8,29–33]. A total of 41 predictors were included in the analysis, including clinical conditions, comorbid conditions based on Charlson’s comorbidity index, demographic variables, and smoking status.

To investigate the impact of risk factors on the progression from MCI to AD/ADRD in the presence of a competing risk of death, we utilized the illness–death model [19]. Our analysis focused on the time to AD/ADRD (*T*_1_) and the time to death (*T*_2_), which may be correlated. It is important to note that assuming independence between *T*_1_ and *T*_2_ and separately applying the Cox model to each time-to-event may introduce bias to the results due to not fully accounting for correlation between *T*_1_ and *T*_2_. To address this, we applied the illness–death model to jointly model *T*_1_ and *T*_2_, accounting for their correlation and the possibility of *T*_1_ influencing the occurrence of *T*_2_.

**Figure 3.**
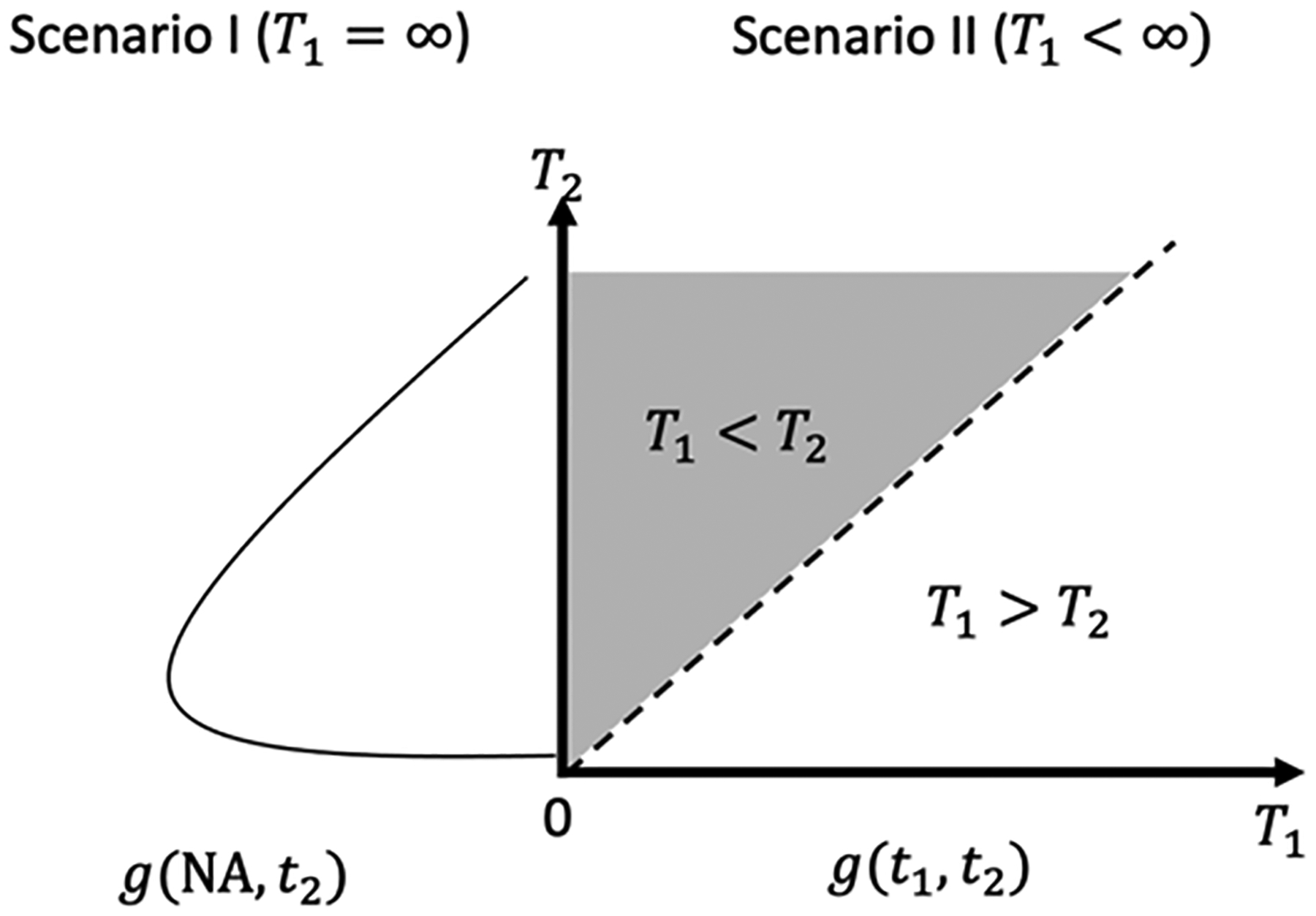
Illustration for the joint distribution of *T*_1_ and *T*_2_.

The model assumes that *T*_1_ and *T*_2_ are semi-competing risks, where an individual can experience one event (AD/ADRD) while remaining at risk for the other event (death). We assumed that AD/ADRD is the non-terminal event and death is the terminal event. In Figure 3, we illustrate the joint distribution of *T*_1_ and *T*_2_. Under Scenario I, only the death event is observed, resulting in the marginal distribution of *T*_2_ when *T*_1_ approaches infinity. Therefore, we assume that the hazard function for death is proportional over time and the censoring process is non-informative. Under Scenario II, both AD/ADRD and death events are observed, with the support of the joint distribution in the upper wedge of the plot (*T*_1_ < *T*_2_) since the time to death always occurred after the time to AD/ADRD. In this scenario, we assume that the risk of AD/ADRD and death are correlated, the hazard functions for both events are proportional over time, and the censoring process is non-informative. These assumptions are crucial for ensuring the validity of the model and obtaining accurate estimates. By utilizing this model, we obtained a more comprehensive understanding of the relationship between AD/ADRD and death and identified potential risk factors for each event.

The illness–death model uses the following equations:

(1) $$h_{1}\left(t_{1}|\alpha_{i},X_{1}\right)=\alpha_{i}h_{01}\left(t_{1}\right)\text{exp}\left(X_{i}^{T}\beta_{1}\right)t_{1}>0$$

(2) $$h_{2}\left(t_{2}|\alpha_{i},X_{1}\right)=\alpha_{i}h_{02}\left(t_{2}\right)\text{exp}\left(X_{i}^{T}\beta_{2}\right)t_{2}>0$$

(3) $$h_{3}\left(t_{2}|t_{1},\alpha_{i},X_{1}\right)=\alpha_{i}h_{03}\left(t_{2}|t_{1}\right)\text{exp}\left(X_{i}^{T}\beta_{3}\right)0<t_{1}<t_{2},$$

where *α*_*i*_ is a patient-specific frailty parameter, and *X*_*i*_ is covariate for *i*th patient [34–36]. The frailty parameter is a random effect that is used to account for unobserved heterogeneity among patients that could affect their risk of experiencing events of interest. In Equation (1), *h*_1_(*t*_1_) denotes the baseline hazard function of time from MCI to AD/ADRD. *β*_1_ denotes a p-dimension coefficient for AD/ADRD from MCI. We interpret the *j*th component of *β*_1_ as the log of the hazard ratio (HR for one unit increase in that component while adjusting for other components of *X* and *α*_*i*_. Similarly, in Equation (2), we denote the baseline hazard function for death from MCI by *h*_2_(*t*_2_) and interpret the *j*th component of exp(*β*_2_) as the hazard ratio (HR) of death from MCI for one unit increase in that component while holding other components of *X* and *α*_*i*_. The difference between the illness–death model and other competing risks model, such as the Fine-Gray model [21], is that the illness–death model measures the transition-specific hazard from AD/ADRD to death, as death is a terminal event whereas AD/ADRD is a non-terminal event. Thus, the transition is from the AD/ADRD event to the death event and is not irreversible. In Equation (3), we denote the baseline hazard function for transitioning from AD/ADRD to death by *h*_3_(*t*_2_|*t*_1_). The *j*th component of exp(*β*_3_) can be interpreted as the HR of death from AD/ADRD to death for one unit increase in that component while adjusting other components of *X* and *α*_*i*_.

In our study, we assumed that *α*_*i*_ follows a gamma distribution, which is a widely used distribution for modeling random effects in survival analysis. The gamma distribution assumption is based on the characteristic that individual frailties are non-negative and have a skewed distribution, which is a common characteristic of frailties in survival analysis. The frailty parameter *α*_*i*_ accounts for the correlation between the time to AD/ADRD and the time to death and reflects the unobserved patient-specific factors that may influence the risk of experiencing the events of interest. The interpretation of *β*_1_, *β*_2_, and *β*_3_ are thus different from fitting a single Cox model for each transition since it incorporates the patient-specific effect in the model. The Bayesian paradigm is computationally efficient and provides a framework for predicting future outcomes. All data analyses in this paper are conducted using R 4.0.3 package “SemiCompRisks” [37].

## 3. Results

**Figure 4.**
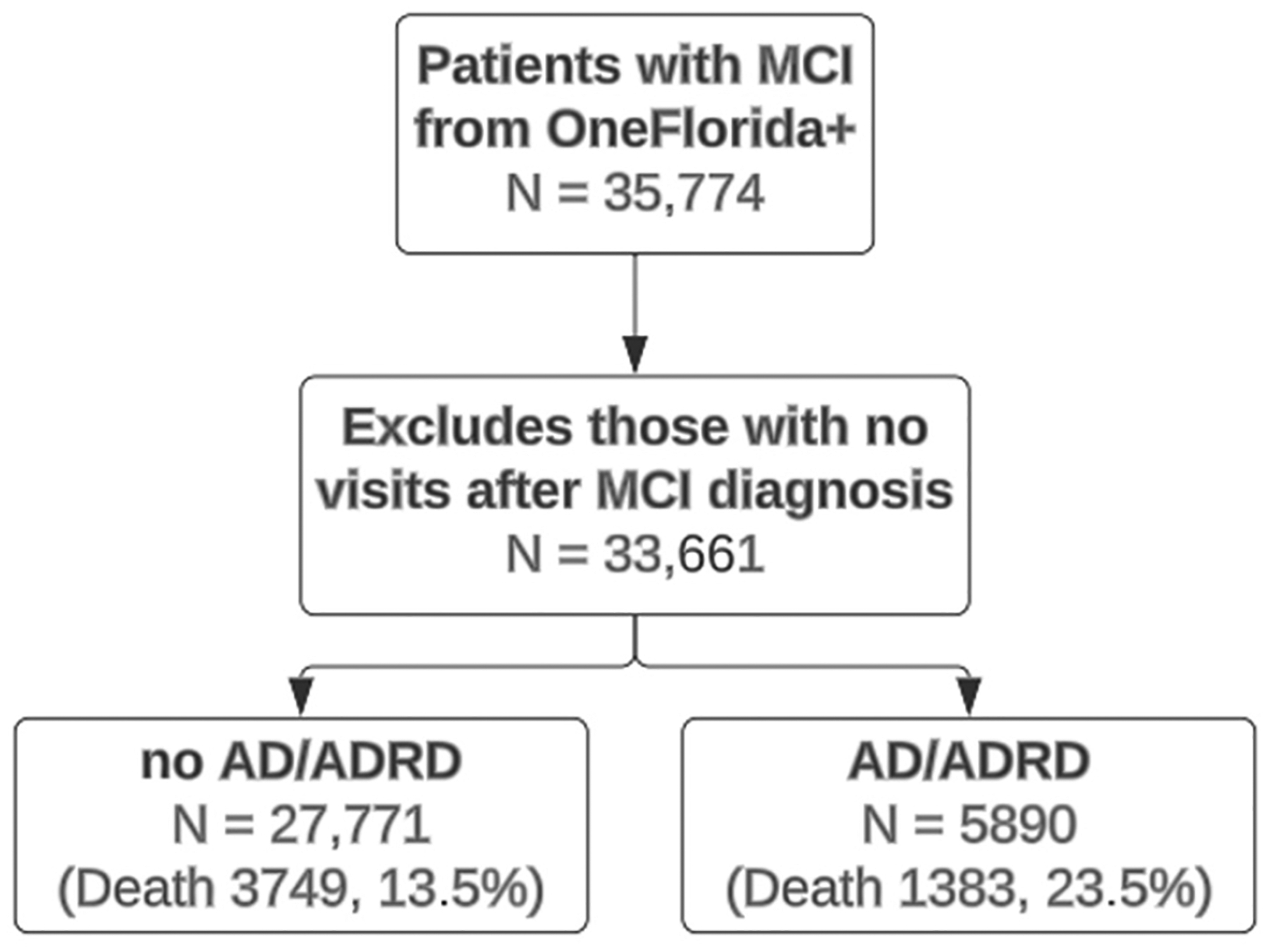
A flow chart of study population selection.

A total of 35,774 patients with MCI were identified from the OneFlorida+ clinical research network. Figure 4 shows a flow diagram of the study cohort. After excluding patients who had no visits after their first MCI diagnosis, 33,661 patients were included in the analysis. Among them, 27,771 did not develop any types of AD/ADRD, while 5890 developed AD/ADRD. Among patients with AD/ADRD, 3214 of them have developed AD, 1268 of them developed vascular dementia, 283 of them developed Lewy body dementia, 105 developed frontotemporal dementia, and 1020 of them have mixed dementia. In terms of the number of death, for patients with no AD/ADRD, 3749 (13.5%) patients died, while for patients with AD/ADRD, 5890 (23.5%) of them died.

The distributions of all baseline characteristics from the predictor extraction window were shown in Table 1 for patients with any type of AD/ADRD vs. those with no AD/ADRD. Compared with those who did not develop AD/ADRD, there were more females (60.1% vs. 52.8%), more Hispanics (25.7% vs. 18.2%), and patients with AD/ADRD also tended to be older (mean age: 74.4 vs. 59.4) and have higher mortality rates (23.5% vs. 13.5%). Both groups have similar BMI, and there are fewer current smokers (9.7% vs. 14.4%) among AD/ADRD patients but more patients with unknown smoking status (61.2% vs. 56.0%). There are also different distributions between AD/ADRD patients and non-AD/ADRD patients in the risk factors we included. In general, AD/ADRD patients tend to have higher frequencies in most diseases except for anxiety, rheumatic disease, liver diseases, hemiplegia or paraplegia, HIV/AIDS, sleep disorder, and visual impairment.

Table 2 shows the hazard ratios for AD/ADRD treating the death as a random censor vs. with consideration of death as a semi-competing risk. Several factors were identified by both models as risk factors for having AD/ADRD, including older age, being Hispanic, having depression, hypertension, diabetes, cerebrovascular diseases, dementia, and stroke. In general, there was not much difference between the two models in terms of hazard ratios (and their corresponding confidence interval) for most included predictors; however, renal diseases, traumatic brain injury, and vision impairment have had larger confidence intervals that are not statistically significant in the model that considered competing risk.

## 4. Discussion

In this study, using a large collection of real-world data from the OneFlorida+ network, we aimed to develop models to predict the conversion from MCI to AD/ADRD with the presence of death as a competing risk. Through this analysis, we have identified several important risk factors for the development of AD/ADRD. We found that patients who have older age, are Hispanics, have depression, diabetes, cerebrovascular diseases, renal disease, or stroke have a higher hazard of having AD/ADRD. These findings are consistent with previous literature [38], indicating the validity of our study. For example, vascular diseases have been linked with an increased risk of AD as the impairments to cerebrovascular network and neurovascular control mechanisms would reduce their abilities to maintain brain activity [39]. History of hypertension, high blood pressure, and heart diseases have all been reported to be associated with a higher risk of AD/ADRD. Diabetes, especially type 2 diabetes (T2D), is also associated with an increased risk of cognitive dysfunction and dementia through mechanisms such as insulin resistance and metabolic syndrome [38,40–42]. Individuals with a history of depression were more likely to develop AD/ADRD later in life, especially those with earlier-life depression. Finally, it has also been reported that Hispanics are more likely to develop AD/ADRD partially because of their higher risk of high blood pressure, heart disease, diabetes, and stroke—all additional risk factors for AD/ADRD [43,44].

In this study, in addition to the standard Cox model, we used a semi-competing risk approach to build the AD/ADRD prediction model. In theory, the use of semi-competing risk models can account for the occurrence of the competing risk event (i.e., death in our case) and its relationship to the primary outcome (AD/ADRD), which improves the accuracy of risk prediction when the two hazards are strongly correlated. In this experiment, the hazard of AD/ADRD (at time *t*) is interpreted as the cause-specific hazard considering a patient-specific effect, i.e., the instantaneous risk of developing AD/ADRD (at time *t*) in the presence of death given not having AD/ADRD or death (up to time *t*). In comparison, the standard Cox model assumes that the only possible outcome is the occurrence of the primary event and does not fully account for the correlation between the primary event and the competing event. Given the complexity of AD/ADRD diseases, this hypothesis is plausible and important to capture in the analytical methods.

The assumptions for the semi-competing risks model are reasonable for this study. First, the independent censoring assumption is untestable [45]. However, we suspect that the event and the censor are conditional independents of the covariates and frailty for the dataset because the risk factors for ADRD, such as age and comorbidities, can also influence the risk of mortality. As we have controlled covariates and considered frailty effects in our model, this helps to account for their influence on the probabilities of death and ADRD. Secondly, regarding the assumption of proportional hazards, we conducted a comprehensive analysis using Schoenfeld residuals and performed formal tests for each covariate included in the data analysis [46]. These evaluations aimed to determine whether the multiplicative relationship holds true. We reported that the majority of covariates (38 of 41 risk factors for time to AD/ADRD and 39 of 41 risk factors for time to death) in our dataset satisfied the proportional hazards assumption. The Schoenfeld residuals exhibited no significant deviations from proportionality, indicating that the hazard ratios for these covariates remain constant over time. Finally, regarding the assumption of frailty distribution, extensive evidence in the statistics literature supports the use of the gamma frailty model in situations where events are positively correlated [26,47,48]. We have thoroughly discussed the justifications for this choice in relation to our dataset and research question. Additionally, we examined our data using Pearson’s correlation to assess the relationship between the occurrence of AD/ADRD and death events, and our analysis revealed a positive and statistically significant correlation coefficient. Consequently, assuming a gamma distribution for the frailty is reasonable. It is worth noting that the semi-competing risks model we employed is also flexible in terms of the choice of frailty assumptions, including the inverse gamma and Gaussian distributions.

However, we did not observe significant differences in the HRs between the semicompeting risks model and the standard Cox model (i.e., significant level > 0.05 for all HR comparisons) except for a few variables. The lack of observed differences between the two models suggests that the two methods perform similarly in predicting AD/ADRD among patients with MCI and that the additional adjustment of the semi-competing regression model did not yield significantly different estimates. It is possible that death may not be a competing risk in the progression between MCI to AD/ADRD, as suggested by the smaller mortality in patients with no AD/ADRD (13.5%) than in patients who developed AD/ADRD (23.5%). Despite the lack of observed differences, it is still important to consider the potential advantages of the semi-competing risk approach, as evident from Table 2; renal disease, traumatic brain injury, sleep disorder, and visual impairment are only statistically significantly associated with AD/ADRD in the model that did not consider death as a competing risk. This suggests that patients with these conditions were at higher risk of developing AD/ADRD compared to those without these conditions only when we treat death as random censoring, which indicates that the association between these predictors and the risk of developing AD/ADRD may be confounded by the presence of death as a semi-competing risk. Therefore, the interpretation of the HRs in our findings should take into account the model used and the presence of death as a semi-competing risk. The HRs obtained from the semi-competing risks model may be useful for predicting the risk of developing AD/ADRD in patients with conditions such as renal disease, traumatic brain injury, sleep disorder, or a visual impairment, and the standard Cox model may not fully capture the association between these predictors and the risk of developing AD/ADRD due to the presence of death as a semi-competing risk.

There are some limitations in this work. First, we only considered death as a potential competing risk; however, MCI patients may also have other significant conditions such as various types of cancers and other types of dementia besides AD/ADRD, especially as they age, which may serve as competing risks. Multiple chronic conditions are highly prevalent in older adults, such as hyperlipidemia, ischemic heart disease, and chronic kidney disease, among others, that are competing risks of AD/ADRD [49]. Nevertheless, our approach can be easier extended to consider multiple competing risks in one model [37,50,51]. Secondly, in this study, we only used the structured data from the OneFlorida+ network where some other important risk factors, such as social determinants of health (SDoH) and clinical narratives, were not readily available and thus not included in our analysis, and there may also be other unmeasured confounding variables that may bias the model. Furthermore, we were only able to model AD/ADRD onset as the outcome. To accurately study the progression of AD/ADRD, we would need to be able to extract and model other intermediate outcomes such as neuropsychological tests (e.g., Mini-Mental State Examination [MMSE]) that are not typically captured in structured EHR either. Advanced natural language processing (NLP) methods can be leveraged to extract additional risk factors and neuropsychological test results that measure disease severity [52] from clinical narratives. It is also worth noting that the progression from MCI to AD/ADRD is a heterogeneous process. Some individuals may progress quickly, while others may not progress at all. There are many factors that can influence the rate and direction of the progression, including age, genetics, comorbidities, lifestyle factors, and other environmental factors. Furthermore, there are multiple subtypes of AD/ADRD. In this study, we grouped five conditions (Alzheimer’s, vascular dementia, Lewy body, frontotemporal dementia, and mixed dementias) together as AD/ADRD, but individuals with different subtypes may present with different patterns of cognitive impairment and neuropathology. As such, a better understanding of the heterogeneity of the progression from MCI to AD/ADRD is essential for the development of personalized treatment and prevention strategies.

In addition to the above-mentioned improvement to the methodology, potential future work in this area could focus on further validating and improving the semi-competing risk model by incorporating additional predictors or exploring different modeling approaches. Additionally, it may be valuable to explore the impact of different types of competing events on the prediction of AD/ADRD, as well as develop and evaluate interventions aimed at reducing the risk of both AD/ADRD and death in this population. Furthermore, it may be beneficial to investigate the generalizability of the findings to other populations and healthcare settings.

While our study developed a novel semi-competing risks regression model to predict the risk of AD/ADRD in individuals with MCI, we did not report the precision and sensitivity of the model in our current findings. We acknowledge that these performance metrics are important for evaluating the predictive ability of the model and are of great interest to the research community. However, due to the limitations of this current study, we were unable to include these results in our analysis. We plan to further investigate the precision and sensitivity of the model in future research and will report our findings in the discussion section (Section 4) of our paper. We believe that this future work will provide valuable insights into the performance of our model and its potential use in clinical practice.

## 5. Conclusions

In this work, using large collections of real-world clinical data from the OneFlorida+ Clinical Research Consortium, we identified a number of risk factors for AD/ADRD, which are consistent with the literature. We considered death as a competing risk and fitted a semi-competing risks model in addition to the standard Cox model. However, we did not observe significant differences from the semi-competing risks model, which suggests that in this specific study, the traditional Cox regression model may be a sufficient and appropriate approach for predicting the occurrence of AD/ADRD in the presence of the competing risk of death among MCI patients. However, further research may be warranted to investigate the performance of semi-competing regression models in other settings and populations.

## Figures and Tables

**Table 1.** Baseline characteristics of the study population.

	No AD/ADRD	Developed AD/ADRD
	(*N* = 27,771)	(*N* = 5890)
**Sex**		
Female	14,654 (52.8%)	3538 (60.1%)
Male	13,117 (47.2%)	2352 (39.9%)
**Race/ethnicity**		
Hispanic	5065 (18.2%)	1515 (25.7%)
NHB	4328 (15.6%)	776 (13.2%)
NHW	12,008 (43.2%)	2577 (43.8%)
Other	1266 (4.6%)	200 (3.4%)
Unknown	5104 (18.4%)	822 (14.0%)
**Age**		
Mean (SD)	59.4 (21.2)	74.4 (12.2)
**Smoking**		
Current smoker	4007 (14.4%)	569 (9.7%)
Former smoker	4995 (18.0%)	1103 (18.7%)
Never smoker	3221 (11.6%)	615 (10.4%)
Unknown	15,548 (56.0%)	3603 (61.2%)
**BMI**		
Mean (SD)	27.4 (6.72)	26.9 (5.46)
**Death**		
Mean (SD)	3749 (13.5%)	1383 (23.5%)
**Anxiety**	9673 (34.8%)	1895 (32.2%)
**Apathy**	32 (0.1%)	6 (0.1%)
**Depression**	12,163 (43.8%)	2653 (45.0%)
**Hypertension**	17,907 (64.5%)	4588 (77.9%)
**Diabetes**	9115 (32.8%)	2386 (40.5%)
**Cerebrovascular diseases**	8088 (29.1%)	2313 (39.3%)
**Cardiovascular diseases**	22,025 (79.3%)	5103 (86.6%)
**Atrial fibrillation**	3266 (11.8%)	982 (16.7%)
**Hypercholesterolemia**	4214 (15.2%)	1081 (18.4%)
**Myocardial infarction**	2303 (8.3%)	601 (10.2%)
**Congestive heart failure**	4559 (16.4%)	1212 (20.6%)
**Peripheral vascular disease**	5280 (19.0%)	1446 (24.6%)
**Cerebrovascular disease**	6964 (25.1%)	2047 (34.8%)
**Chronic pulmonary disease**	8540 (30.8%)	1825 (31.0%)
**Rheumatic disease**	1263 (4.5%)	241 (4.1%)
**Peptic ulcer disease**	1060 (3.8%)	234 (4.0%)
**Mild liver disease**	3348 (12.1%)	503 (8.5%)
**Diabetes without chronic complication**	8170 (29.4%)	2131 (36.2%)
**Diabetes with chronic complication**	3608 (13.0%)	936 (15.9%)
**Hemiplegia or paraplegia**	2166 (7.8%)	351 (6.0%)
**Renal disease**	4363 (15.7%)	1189 (20.2%)
**Any malignancy**	3158 (11.4%)	553 (9.4%)
**Moderate or severe liver disease**	513 (1.8%)	64 (1.1%)
**Metastatic solid tumor**	750 (2.7%)	81 (1.4%)
**AIDS/HIV**	562 (2.0%)	33 (0.6%)
**Obesity**	7961 (28.7%)	1235 (21.0%)
**hyperlipidemia**	12,375 (44.6%)	3134 (53.2%)
**Stroke**	13,570 (48.9%)	3376 (57.3%)
**Traumatic brain injury**	6088 (21.9%)	1881 (31.9%)
**Sleep disorder**	3153 (11.4%)	583 (9.9%)
**Periodontitis**	6323 (22.8%)	1177 (20.0%)
**Alcohol use disorder**	225 (0.8%)	32 (0.5%)
**Exercise**	2554 (9.2%)	383 (6.5%)
**Visual impairment**	754 (2.7%)	89 (1.5%)
**Hearing impairment**	453 (1.6%)	112 (1.9%)

SD: standard deviation. AIDS/HIV: acquired immunodeficiency syndrome/human immunodeficiency virus.

**Table 2.** Hazard ratios for the occurrence of Alzheimer’s disease and AD-related dementias (AD/ADRD) with treating death as random censoring vs. with consideration of death as a semicompeting risk.

Variable	Hazard Ratio (HR)	
	Treating Death as Random Censoring	Considering Death as a Semi-Competing Risk
Age	1.054 (1.052, 1.057) *	1.049 (1.047, 1.054) *
Sex (ref = Male)	0.958 (0.907, 1.012)	0.969 (0.908, 1.014)
Race/ethnicity (ref = NHW)		
Hispanic	1.257 (1.178, 1.341) *	1.233 (1.154, 1.317) *
NHB	1.040 (0.957, 1.113)	1.014 (0.934, 1.109)
Other	0.702 (0.606, 0.814) *	0.721 (0.625, 0.827) *
Unknown	0.826 (0.761, 0.896) *	0.838 (0.774, 0.916) *
Anxiety	1.027 (0.964, 1.093)	0.965 (0.903, 1.016)
Depression	1.205 (1.136, 1.278) *	1.096 (1.027, 1.165) *
Hypertension	1.071 (0.975, 1.177)	1.047 (0.941, 1.139)
Diabetes	1.146 (1.006, 1.305) *	1.170 (1.034, 1.329) *
Cerebrovascular diseases	1.187 (1.068, 1.322) *	1.287 (1.158, 1.450) *
Cardiovascular diseases	0.928 (0.829, 1.040)	0.938 (0.850, 1.026)
Atrial fibrillation	0.972 (0.901, 1.048)	0.979 (0.906, 1.074)
Hypercholesterolemia	0.987 (0.918, 1.061)	1.000 (0.930, 1.087)
Myocardial infarction	1.008 (0.919, 1.106)	1.056 (0.966, 1.156)
Congestive heart failure	0.995 (0.922, 1.074)	0.941 (0.869, 1.012)
Peripheral vascular disease	1.008 (0.941, 1.079)	0.984 (0.923, 1.066)
Cerebrovascular disease	1.044 (0.923, 1.182)	0.909 (0.899, 1.020)
Chronic pulmonary disease	0.980 (0.921, 1.044)	0.950 (0.899, 1.020)
Rheumatic disease	0.809 (0.710, 0.923) *	0.817 (0.725, 0.938) *
Peptic ulcer disease	1.045 (0.912. 1.204)	1.045 (0.912. 1.204)
Mild liver disease	0.875 (0.792, 0.967) *	0.894 (0.804, 0.990) *
Diabetes without chronic complication	0.974 (0.852, 1.113)	0.935 (0.824, 1.060)
Diabetes with chronic complication	1.012 (0.927, 1.104)	0.995 (0.908, 1.084)
Hemiplegia or paraplegia	1.041 (0.929, 1.166)	1.022 (0.907, 1.143)
Renal disease	1.096 (1.020, 1.178) *	1.031 (0.907, 1.143)
Any malignancy	0.815 (0.742, 0.894) *	0.822 (0.748, 0.897) *
Moderate or severe liver disease	0.985 (0.761, 1.275)	0.963 (0.742, 0.897) *
Metastatic solid tumor	0.865 (0.687, 1.090)	0.894 (0.730, 1.138)
AIDS/HIV	0.502 (0.356, 0.709) *	0.471 (0.335, 0.660) *
Obesity	0.811 (0.756, 0.871) *	0.819 (0.763, 0.886) *
hyperlipidemia	0.961 (0.900, 1.026)	0.941 (0.866, 1.015)
Stroke	1.084 (1.011, 1.162) *	1.184 (1.085, 1.310) *
Traumatic brain injury	1.110 (1.015, 1.214) *	0.998 (0.891, 1.084)
Sleep disorder	0.908 (0.847, 0.973) *	0.897 (0.838, 0.964) *
Periodontitis	1.095 (0.773, 1.555)	1.195 (0.825, 1.675)
Alcohol use	1.106 (0.988, 1.238)	1.099 (0.973, 1.224)
Exercise	0.995 (0.806, 1.229)	1.035 (0.860, 1.345)
Visual impairment	1.211 (1.002, 1.463) *	1.128 (0.934, 1.329)
Hearing impairment	0.861 (0.785, 0.944) *	0.827 (0.752, 0.909) *

* Indicate a *p*-value < 0.05 that is considered statistically significant.

NHW: non-Hispanic White; NHB: non-Hispanic Black. AIDS/HIV: acquired immunodeficiency syndrome/human immunodeficiency virus.

## Data Availability

The data used in this study available upon request through the One Florida+ Clinical Research Network (https://onefloridaconsortium.org/).

## References

[1] Alzheimer’s Association. 2022 Alzheimer’s disease facts and figures. Alzheimer’s Dement 2022, 18, 700–789. 35289055 10.1002/alz.12638

[2] Wong W Economic Burden of Alzheimer Disease and Managed Care Considerations. Suppl. Featured Publ 2020, 26, S177–S183. Available online: https://cdn.sanity.io/files/0vv8moc6/ajmc/732db911e3e68ecd4cdb59c9b2ed2556c903e975.pdf/AJMC_ACE0178_Alzheimer__Web.pdf (accessed on 20 April 2022). 10.37765/ajmc.2020.88482 32840331

[3] Lam B; Masellis M; Freedman M; Stuss DT; Black SE Clinical, imaging, and pathological heterogeneity of the Alzheimer’s disease syndrome. Alzheimer’s Res. Ther 2013, 5, 1. 23302773 10.1186/alzrt155 PMC3580331

[4] Goyal D; Tjandra D; Migrino RQ; Giordani B; Syed Z; Wiens J Characterizing heterogeneity in the progression of Alzheimer’s disease using longitudinal clinical and neuroimaging biomarkers. Alzheimer’s Dement. Diagn. Assess. Dis. Monit 2018, 10, 629–637. 10.1016/j.dadm.2018.06.007 PMC6234900 30456290

[5] Petersen RC; Smith GE; Waring SC; Ivnik RJ; Tangalos EG; Kokmen E Mild cognitive impairment: Clinical characterization and outcome. Arch. Neurol 1999, 56, 303–308. 10190820 10.1001/archneur.56.3.303

[6] Tábuas-Pereira M; Baldeiras I; Duro D; Santiago B; Ribeiro MH; Leitão MJ; Oliveira C; Santana I Prognosis of Early-Onset vs. Late-Onset Mild Cognitive Impairment: Comparison of Conversion Rates and Its Predictors. Geriatrics 2016, 1, 11. 31022805 10.3390/geriatrics1020011 PMC6371125

[7] Davis M; Connell OT; Johnson S; Cline S; Merikle E; Martenyi F; Simpson K Estimating Alzheimer’s Disease Progression Rates from Normal Cognition Through Mild Cognitive Impairment and Stages of Dementia. Curr. Alzheimer Res 2018, 15, 777–788. 29357799 10.2174/1567205015666180119092427 PMC6156780

[8] Farias ST; Mungas D; Reed BR; Harvey D; DeCarli C Progression of mild cognitive impairment to dementia in clinic- vs community-based cohorts. Arch. Neurol 2009, 66, 1151–1157. 19752306 10.1001/archneurol.2009.106 PMC2863139

[9] Bozoki A; Giordani B; Heidebrink JL; Berent S; Foster NL Mild cognitive impairments predict dementia in nondemented elderly patients with memory loss. Arch. Neurol 2001, 58, 411–416. 11255444 10.1001/archneur.58.3.411

[10] Plassman BL; Langa KM; Fisher GG; Heeringa SG; Weir DR; Ofstedal MB; Burke JR; Hurd MD; Potter GG; Rodgers WL Prevalence of cognitive impairment without dementia in the United States. Ann. Intern. Med 2008, 148, 427–434. 18347351 10.7326/0003-4819-148-6-200803180-00005 PMC2670458

[11] Kumar S; Oh I; Schindler S; Lai AM; Payne PRO; Gupta A Machine learning for modeling the progression of Alzheimer disease dementia using clinical data: A systematic literature review. JAMIA Open 2021, 4, ooab052. 34350389 10.1093/jamiaopen/ooab052 PMC8327375

[12] Grueso S; Viejo-Sobera R Machine learning methods for predicting progression from mild cognitive impairment to Alzheimer’s disease dementia: A systematic review. Alzheimer’s Res. Ther 2021, 13, 162. 34583745 10.1186/s13195-021-00900-w PMC8480074

[13] Rowe TW; Katzourou IK; Stevenson-Hoare JO; Bracher-Smith MR; Ivanov DK; Escott-Price V Machine learning for the life-time risk prediction of Alzheimer’s disease: A systematic review. Brain Commun 2021, 3, fcab246. 34805994 10.1093/braincomms/fcab246 PMC8598986

[14] Sherman RE; Anderson SA; Dal Pan GJ; Gray GW; Gross T; Hunter NL; LaVange L; Marinac-Dabic D; Marks PW; Robb MA; Real-world evidence—What is it and what can it tell us? N. Engl. J. Med 2016, 375, 2293–2297. 27959688 10.1056/NEJMsb1609216

[15] Rajkomar A; Oren E; Chen K; Dai AM; Hajaj N; Hardt M; Liu PJ; Liu X; Marcus J; Sun M Scalable and accurate deep learning with electronic health records. NPJ Digit. Med 2018, 1, 18. 31304302 10.1038/s41746-018-0029-1 PMC6550175

[16] Tomašev N; Glorot X; Rae JW; Zielinski M; Askham H; Saraiva A; Mottram A; Meyer C; Ravuri S; Protsyuk I; A clinically applicable approach to continuous prediction of future acute kidney injury. Nature 2019, 572, 116–119. 31367026 10.1038/s41586-019-1390-1 PMC6722431

[17] Xu Z; Chou J; Zhang XS; Luo Y; Isakova T; Adekkanattu P; Ancker JS; Jiang G; Kiefer RC; Pacheco JA; Identifying sub-phenotypes of acute kidney injury using structured and unstructured electronic health record data with memory networks. J. Biomed. Inform 2020, 102, 103361. 31911172 10.1016/j.jbi.2019.103361

[18] Zhang X; Chou J; Liang J; Xiao C; Zhao Y; Sarva H; Henchcliffe C; Wang F Data-Driven Subtyping of Parkinson’s Disease Using Longitudinal Clinical Records: A Cohort Study. Sci. Rep 2019, 9, 797. 30692568 10.1038/s41598-018-37545-z PMC6349906

[19] Zhang P; Wang F; Hu J; Sorrentino R Towards personalized medicine: Leveraging patient similarity and drug similarity analytics. AMIA Summits Transl. Sci. Proc 2014, 2014, 132–136. 25717413 PMC4333693

[20] Wang F; Preininger A AI in Health: State of the Art, Challenges, and Future Directions. Yearb. Med. Inform 2019, 28, 16–26. 31419814 10.1055/s-0039-1677908 PMC6697503

[21] Fine JP; Gray RJ A Proportional Hazards Model for the Subdistribution of a Competing Risk. J. Am. Stat. Assoc 1999, 94, 496–509.

[22] Hou Y; Dan X; Babbar M; Wei Y; Hasselbalch SG; Croteau DL; Bohr VA Ageing as a risk factor for neurodegenerative disease. Nat. Rev. Neurol 2019, 15, 565–581. 31501588 10.1038/s41582-019-0244-7

[23] World Report on Ageing and Health. 2015. Available online: https://www.who.int/publications/i/item/9789241565042 (accessed on 21 April 2023).

[24] Christensen K; Doblhammer G; Rau R; Vaupel JW Ageing populations: The challenges ahead. Lancet 2009, 374, 1196–1208. 19801098 10.1016/S0140-6736(09)61460-4 PMC2810516

[25] Costantino S; Paneni F; Cosentino F Ageing, metabolism and cardiovascular disease. J. Physiol 2016, 594, 2061–2073. 26391109 10.1113/JP270538 PMC4933114

[26] Fine JP; Jiang H; Chappell R On semi-competing risks data. Biometrika 2001, 88, 907–919.

[27] Hogan WR; Shenkman EA; Robinson T; Carasquillo O; Robinson PS; Essner RZ; Bian J; Lipori G; Harle C; Magoc T The OneFlorida Data Trust: A centralized, translational research data infrastructure of statewide scope. J. Am. Med. Inform. Assoc 2021, 29, 686–693. 10.1093/jamia/ocab221 PMC8922180 34664656

[28] Bian J; Loiacono A; Sura A; Mendoza Viramontes T; Lipori G; Guo Y; Shenkman E; Hogan W Implementing a hash-based privacy-preserving record linkage tool in the OneFlorida clinical research network. JAMIA Open 2019, 2, 562–569. 32025654 10.1093/jamiaopen/ooz050 PMC6994009

[29] van der Flier WM; Scheltens P Epidemiology and risk factors of dementia. J. Neurol. Neurosurg. Psychiatry 2005, 76 (Suppl. S5), v2–v7. 16291918 10.1136/jnnp.2005.082867 PMC1765715

[30] Azad NA; Al Bugami M; Loy-English I Gender differences in dementia risk factors. Gend. Med 2007, 4, 120–129. 17707846 10.1016/s1550-8579(07)80026-x

[31] Tariq S; Barber PA Dementia risk and prevention by targeting modifiable vascular risk factors. J. Neurochem 2018, 144, 565–581. 28734089 10.1111/jnc.14132

[32] Lindsay J; Laurin D; Verreault R; Hébert R; Helliwell B; Hill GB; McDowell I Risk factors for Alzheimer’s disease: A prospective analysis from the Canadian Study of Health and Aging. Am. J. Epidemiol 2002, 156, 445–453. 12196314 10.1093/aje/kwf074

[33] Imtiaz B; Tolppanen AM; Kivipelto M; Soininen H Future directions in Alzheimer’s disease from risk factors to prevention. Biochem. Pharmacol 2014, 88, 661–670. 24418410 10.1016/j.bcp.2014.01.003

[34] Xu J; Kalbfleisch JD; Tai B Statistical analysis of illness-death processes and semicompeting risks data. Biometrics 2010, 66, 716–725. 19912171 10.1111/j.1541-0420.2009.01340.x

[35] Lee KH; Haneuse S; Schrag D; Dominici F Bayesian Semi-parametric Analysis of Semi-competing Risks Data: Investigating Hospital Readmission after a Pancreatic Cancer Diagnosis. J. R. Stat. Society Ser. C Appl. Stat 2015, 64, 253–273. 10.1111/rssc.12078 PMC4427057 25977592

[36] Lee KH; Dominici F; Schrag D; Haneuse S Hierarchical models for semi-competing risks data with application to quality of end-of-life care for pancreatic cancer. J. Am. Stat. Assoc 2016, 111, 1075–1095. 28303074 10.1080/01621459.2016.1164052 PMC5347153

[37] Alvares D; Haneuse S; Lee C; Lee KH SemiCompRisks: An R Package for the Analysis of Independent and Cluster-correlated Semi-competing Risks Data. R J 2019, 11, 376–400. 33604061 10.32614/rj-2019-038 PMC7889044

[38] Baumgart M; Snyder HM; Carrillo MC; Fazio S; Kim H; Johns H Summary of the evidence on modifiable risk factors for cognitive decline and dementia: A population-based perspective. Alzheimer’s Dement 2015, 11, 718–726. 26045020 10.1016/j.jalz.2015.05.016

[39] Iadecola C Neurovascular regulation in the normal brain and in Alzheimer’s disease. Nat. Rev. Neurosci 2004, 5, 347–360. 15100718 10.1038/nrn1387

[40] Biessels GJ; Kappelle LJ; Utrecht Diabetic Encephalopathy Study Group. Increased risk of Alzheimer’s disease in Type II diabetes: Insulin resistance of the brain or insulin-induced amyloid pathology? Biochem. Soc. Trans 2005, 33, 1041–1044. 16246041 10.1042/BST0331041

[41] Chatterjee S; Peters SA; Woodward M; Mejia Arango S; Batty GD; Beckett N; Beiser A; Borenstein AR; Crane PK; Haan M; Type 2 Diabetes as a Risk Factor for Dementia in Women Compared With Men: A Pooled Analysis of 2.3 Million People Comprising More Than 100,000 Cases of Dementia. Diabetes Care 2016, 39, 300–307. 26681727 10.2337/dc15-1588 PMC4722942

[42] Baglietto-Vargas D; Shi J; Yaeger DM; Ager R; LaFerla FM Diabetes and Alzheimer’s disease crosstalk. Neurosci. Biobehav. Rev 2016, 64, 272–287. 26969101 10.1016/j.neubiorev.2016.03.005

[43] Vega IE; Cabrera LY; Wygant CM; Velez-Ortiz D; Counts SE Alzheimer’s Disease in the Latino Community: Intersection of Genetics and Social Determinants of Health. J. Alzheimer’s Dis 2017, 58, 979–992. 28527211 10.3233/JAD-161261 PMC5874398

[44] Stickel A; McKinnon A; Ruiz J; Grilli MD; Ryan L The impact of cardiovascular risk factors on cognition in Hispanics and non-Hispanic whites. Learn. Mem 2019, 26, 235–244. 31209118 10.1101/lm.048470.118 PMC6581002

[45] Klein JP; Moeschberger ML Survival Analysis; Springer: New York, NY, USA, 1997.

[46] Grambsch PM; Therneau TM Proportional hazards tests and diagnostics based on weighted residuals. Biometrika 1994, 81, 515–526.

[47] Clayton DG A model for association in bivariate life tables and its application in epidemiological studies of familial tendency in chronic disease incidence. Biometrika 1978, 65, 141–151.

[48] Oakes D Semiparametric inference in a model for association in bivanate survival data. Biometrika 1986, 73, 353–361.

[49] He Z; Bian J; Carretta HJ; Lee J; Hogan WR; Shenkman E; Charness N Prevalence of multiple chronic conditions among older adults in Florida and the United States: Comparative analysis of the OneFlorida data trust and National Inpatient Sample. J. Med. Internet Res 2018, 20, e137. 29650502 10.2196/jmir.8961 PMC5920146

[50] Dignam JJ; Zhang Q; Kocherginsky M The use and interpretation of competing risks regression models. Clin. Cancer Res 2012, 18, 2301–2308. 22282466 10.1158/1078-0432.CCR-11-2097 PMC3328633

[51] Zhang Z Survival analysis in the presence of competing risks. Ann. Transl. Med 2017, 5, 47. 28251126 10.21037/atm.2016.08.62 PMC5326634

[52] Chen Z; Zhang H; Yang X; Wu S; He X; Xu J; Guo J; Prosperi M; Wang F; Xu H; Assess the documentation of cognitive tests and biomarkers in electronic health records via natural language processing for Alzheimer’s disease and related dementias. Int. J. Med. Inform 2023, 170, 104973. 36577203 10.1016/j.ijmedinf.2022.104973 PMC11325083

